# Application of Mass Multivariate Analysis on Neuroimaging Data Sets for Precision Diagnostics of Depression

**DOI:** 10.3390/diagnostics12020469

**Published:** 2022-02-12

**Authors:** Rositsa Paunova, Sevdalina Kandilarova, Anna Todeva-Radneva, Adeliya Latypova, Ferath Kherif, Drozdstoy Stoyanov

**Affiliations:** 1 Department of Psychiatry and Medical Psychology, Medical University Plovdiv, 4002 Plovdiv, Bulgaria; rositsa.paunova@mu-plovdiv.bg (R.P.); sevdalina.kandilarova@mu-plovdiv.bg (S.K.); anna.todeva@mu-plovdiv.bg (A.T.-R.); 2 Research Institute, Medical University Plovdiv, 4002 Plovdiv, Bulgaria; 3 Centre for Research in Neuroscience, Department of Clinical Neurosciences, CHUV—UNIL, 1011 Lausanne, Switzerland; adeliya.latypova@chuv.ch (A.L.); ferah.kherif@unil.ch (F.K.)

**Keywords:** mass multivariate analysis, neuroimaging, depression, schizophrenia

## Abstract

We used the Mass Multivariate Method on structural, resting-state, and task-related fMRI data from two groups of patients with schizophrenia and depression in order to define several regions of significant relevance to the differential diagnosis of those conditions. The regions included the left planum polare (PP), the left opercular part of the inferior frontal gyrus (OpIFG), the medial orbital gyrus (MOrG), the posterior insula (PIns), and the parahippocampal gyrus (PHG). This study delivered evidence that a multimodal neuroimaging approach can potentially enhance the validity of psychiatric diagnoses. Structural, resting-state, or task-related functional MRI modalities cannot provide independent biomarkers. Further studies need to consider and implement a model of incremental validity combining clinical measures with different neuroimaging modalities to discriminate depressive disorders from schizophrenia. Biological signatures of disease on the level of neuroimaging are more likely to underpin broader nosological entities in psychiatry.

## 1. Introduction

The World Health Organization has noted a significant increase in the global prevalence of mental illness including conditions such as mood disorders and schizophrenia (SCH). These disorders evoke an enormous social burden, since they cause chronic disability, and also have a high comorbidity rate as well as pernicious consequences not only for the individual but also for their immediate family. Yet, their diagnostic and therapeutic framework has not been marked by scientific advancements to the same degree as that observed for other medical disciplines. In particular, there are many discrepancies and controversies in the currently existing *ex convention* taxonomies [1]. Therefore, it is of vital necessity to provide pro-innovative methodological tools that can assist in the definition of the underlying mechanisms of psychiatric disease and thereby redefine medical classifications applied in psychiatry [2].

A major predicament in contemporary Psychiatry remains the disconformity between the traditional evidence-based medical framework and the inconsistent neuroscientific explanation of the etiopathophysiology of mental illness [3]. Despite the tremendous technological progress in medical diagnostics, psychiatrists still rely mostly on clinical observation and patient evaluation in order to specify a mental disorder and determine a therapeutic pathway. Furthermore, the categorization of psychiatric nosology is not only biologically invalidated but also inadequate to explicitly discern conditions with overlapping pathological behavioral patterns [4,5].

One of the methods which can be the foundation of such a tool is Magnetic Resonance Imaging (MRI). It allows the exploration of multiple imaging modalities, thus potentiating not only the study of different pathophysiological mechanisms but also the discovery of diagnostic and/or prognostic biomarkers [6]. However, the hitherto determined findings in mental disorders, Major Depressive Disorder (MDD) and SCH in particular, have been heterogeneous and inconsistent [7]. This is due to the variety of existing research limitations such as the undefined norm in Psychiatry, the lack of standardized data processing methodology, unavoidable confounding factors, etc. [8].

In our previous studies, we established the sensitivity of the various modalities of MRI in the comparison of depressed patients and healthy controls (HC). We identified structural, functional (resting-state connectivity), and functional (diagnostic task- related) differences in brain structure and function [9,10,11].

On a structural level by means of voxel-based morphometry analysis, we determined a significant grey matter volume (GMV) reduction in several regions in the left (medial frontal and anterior cingulate cortex (ACC)) and in the right (middle frontal gyrus (MFG), medial orbital gyrus, inferior frontal gyrus (IFG), middle temporal gyrus) hemisphere in patients with depression compared to healthy controls [9]. The role of grey matter aberrations in the fronto-limbic regions in the pathophysiology of MDD has also been confirmed in a recent meta-analysis [12].

On a functional level of resting state, using spectral dynamic causal modeling to explore the brain connectivity patterns in depression, our research team found a significantly decreased strength of the connection between the anterior insula (AI) and the middle frontal gyrus (MFG) in patients with depressive syndrome in comparison with healthy individuals, as well as a significant additional connection from the amygdala to the AI, which was found in the patient sample but not in the HC group [10].

With task-related functional MRI, we found significant differences in the BOLD patterns during the implementation of a task between patients with a depressive syndrome and healthy controls. The task itself consisted in the application of self-assessment scales (namely, the von Zerssen self-assessment depression scale as a diagnostically specific condition, and items from a questionnaire about general interests and likes as a diagnostically neutral condition) during MRI scanning (with an MRI protocol including a structural, resting-state sequence and a block design task) [11]. The results obtained from contrasting the diagnostically specific versus the neutral blocks yielded residual activations in brain regions such as the left middle frontal gyrus, the middle temporal gyrus, the right pre- and postcentral gyrus, and the triangular part of the left inferior frontal gyrus in the patient group as opposed to the HC.

These results encouraged us to continue our endeavor in defining the biological underpinnings of depression, and we expanded the paradigm by adding blocks comprised of paranoia-specific items from the von Zerssen’s paranoid subscale. Considering the phenomenological overlap between the clinical presentation of depression and the negative symptoms of SCH [13], we recognized the comparison between the depressive and the paranoid syndromes as a viable tool for exploring the specificity of the paradigm. However, the direct contrast between the depression-specific and paranoia-specific blocks did not result in the expected between-group differentiating residual signal, but did show promising findings at the within-group level [14].

In order to resolve this issue, we decided to approach the problem not only by implementing an innovative paradigm design but also by analyzing the data by means of machine learning [11,14,15]. Our method of choice was the Multivariate Linear Model (MLM), since it can be employed in the analysis of various neuroimaging techniques [16,17] and allows the processing of multidimensional data sets [18].

In an earlier publication, we used MLM to investigate the differentiating capacity of several MRI modalities (namely, structural, resting-state, and functional) combined into principal components (PC) in order to define and discriminate between two syndromes: paranoid and depressive [19]. We defined three signatures comprising different correlations of the three modalities. It was demonstrated that the regions with the highest discriminative power belonged to the Effort Mode Network (EMN) and the Default Mode Network (DMN), as well as the Limbic System. Considering the role of these loci in cognition, emotion processing, decision-making, etc., our findings appear to be consistent with the hitherto acquired knowledge on depression and psychosis, since the phenomenology of both conditions includes dysfunction in some or all of the aforementioned domains [20,21,22].

These findings encouraged the expansion of this method towards a more explicit definition of the active neural domains with a capacity to differentiate the studied paranoid and depressive syndromes. Therefore, we decided to extend the mass-univariate approach hypothesizing that it would allow the determination of a multivariate signature comprising all existing modalities for each region. Having in mind our previous investigations with case–control design, which revealed certain patterns of unimodal (univariate) differences between patients and control subjects, the clinical and scientific rationale of the present study was to determine potential multimodal brain signatures of mental disorders on the level of differential diagnosis of depression, i.e., its specificity.

The Kraepelinian dichotomy asserts manic depression (now incorporated in the category of mood disorders) as a fundamentally independent entity from dementia praecox (which evolved to be the primary concept of psychosis) [23]. However, contemporary science has challenged this historical landmark via researching converse concepts such as the comorbid approach (explaining the similar phenomenology of the depressive syndrome and the negative symptoms of schizophrenia), the existence of common pathophysiological pathways, the dimensional approach which sets the depressive and psychotic syndromes as the opposite ends of the same spectrum, etc. [24] Therefore, we made the decision to determine the specificity of our paradigm by including patients with psychosis. Subsequently, various multimodal signatures were to be explored in order to allow a validated differentiation of the depressive syndrome.

## 2. Materials and Methods

### 2.1. Participants

For the current study, 44 patients were recruited either with a current psychotic (*n* = 19, mean age 39.3 ± 14.8 y, 9 males) or a depressive (*n* = 25, mean age 44.2 ± 12.1 y, 9 males) episode. The psychotic patients had a first-axis diagnosis of schizophrenia, whereas the depressed patients were diagnosed with major depressive disorder (*n* = 10, mean age 43.7 ± 13.2 y, 5 males) or bipolar disorder (*n* = 15, mean age 44.5 ± 11.8 y, 4 males). All individuals were assessed by experienced psychiatrists (D.S., S.K.) using the general clinical interview and the structured Mini International Neuropsychiatric Interview (M.I.N.I 6.0) [25] and Clinical Global Impression (CGI) scale [26]. The Montgomery–Åsberg Depression Rating Scale (MADRS) [27] and the Positive and Negative Syndrome Scale (PANSS) [28] were also used. Patients with depression with a total MADRS score of at least 20 were included, as well as psychotic patients with at least 3 on P1 (delusions) or P6 (suspiciousness) PANSS. Both groups had been on stable medication for the past 14 days. Detailed data on the various medications taken are available at https://zenodo.org/record/5865628 (accessed on 1 February 2022), along with the results and code used for the analysis.

We adhered to the following exclusion criteria: age under 18 years or over 65 years, presence of MRI-incompatible metal implants or body grafts (e.g., pacemaker), severe somatic or neurological disease, comorbid mental disorder, (e.g., substance or alcohol use disorder, obsessive compulsive disorder, etc.), and traumatic brain injury with loss of consciousness. Each participant provided a written informed consent in compliance with the Declaration of Helsinki. The study protocol was approved by the University’s Ethics Committee.

### 2.2. Image Acquisition

The scanning protocol was implemented on a 3T MRI system (GE Discovery 750w) with 3 different MRI sequences: high-resolution structural scan (Sag 3D T1 FSPGR sequence), with slice thickness of 1 mm, matrix 256 × 256, TR (relaxation time) of 7.2 ms, TE (echo time) of 2.3 ms, and flip angle 12°, with two functional scans (2D EPI sequence) while resting with the eyes closed—slice thickness 3 mm, 36 slices, matrix 64 × 64, TR—2000 ms, TE, 30 ms, flip angle 90°, 192 volumes—and during the task (see following paragraph)—slice thickness 3 mm, matrix 64 × 64, TR 2000 ms, TE 30 ms, and flip angle 90°, 256 volumes. The functional scan initiated with 5 dummy time series which were automatically excluded.

### 2.3. FMRI Task

The paradigm was designed via E-prime software (Psychology Software Tools, Inc., Sharpsburg, PA, USA) and included 32 s blocks with three different active conditions. Each active block was followed by 20 s block with the rest (off) condition (fixation cross). The stimuli were presented using the Nordic Neuro Lab Visual System. There is a detailed description of the task in our previous work [15,19]; therefore, here we will only summarize it briefly.

All active blocks contained four written statements from the von Zerssen Paranoid-Depression Scale with a duration of 8 s each. There were Depression-Specific (DS) blocks with items such as “I often feel simply miserable”, “I don’t have any feelings anymore”, and Paranoid-Specific (PS) blocks with items such as “Other people constantly follow and control me”. The Diagnostically Neutral (DN) blocks included statements from a general interest and likes questionnaire (e.g., “I like to write books or plays”, “I like to repair household appliances”, etc.). There were four possible answers (“completely true”, “mostly true”, “somewhat true”, “not true”) corresponding to four response buttons (upper left, lower left, lower right, upper right). The description of the possible answers and the respective buttons was presented under each statement. The whole task consisted of four blocks of each type, alternating between the three active conditions and followed by the rest condition (DS__rest__DN__rest__PS__rest…). The participants were instructed to read the statements carefully and to respond with a button press according to their level of agreement. During the rest condition, they had to focus on the fixation cross without thinking of anything in particular.

### 2.4. MRI Data Analysis

#### 2.4.1. Structural Data Analysis—Voxel-Based Morphometry

The analysis of the MRI images was performed using the SPM 12 (Statistical Parametric Mapping, http://www.fil.ion.ucl.ac.uk/spm/ (accessed on 1 February 2022)) toolbox running on MATLAB R2021a for Windows. The preprocessing of the T1 weighted images included segmentation and normalization to standardized MNI space, followed by spatial smoothing with an 8 mm (FWHM) Gaussian kernel. In addition, the total intracranial volume (TIV) was calculated for each subject using the result of the segmentation step.

#### 2.4.2. Task-Related Functional Data Analysis

The functional images first underwent realignment for correction of head motion, co-registration with the high-resolution anatomical image, normalization to MNI (Montreal Neurological Institute) space, and spatial smoothing with an 8 mm full-width-at-half-maximum (FWHM) Gaussian kernel.

After preprocessing, a first-level analysis of the functional times series was conducted using a general linear model (GLM) that included the conditions convolved with a canonical hemodynamic response function. In the GLM model, covariates of no interest included the six rigid body motion correction parameters. In order to summarize the functional brain activity of each individual, we calculated the Individual F-contrasts using the first-level GLM. The Individual F-contrasts compared the active and passive condition in order to generate input for multivariate analyses.

#### 2.4.3. Resting State Data Analysis

For the resting state data after preprocessing (which was the same as for the task-related data), a general linear model (GLM) applied to the time series was conducted for a first-level analysis. Nuisance covariates included the six rigid body motion parameters, average white matter, and cerebrospinal fluid signal time series. The individual residual mean-square images were also used for the analysis. More information can be found in our previous study (see [29]).

### 2.5. Mass Multivariate Analysis

Our multivariate method extended the mass-univariate approach widely used in the field, in which each dependent variable is considered at a time, to take into account multiple variables. Rather than analyzing each modality separately and determining their association with the diagnosis, as we have done so far, we hypothesized that a multivariate signature based on all of the modalities exists for each brain region. The multivariate linear model fitted here aims to identify the combination that maximizes the square difference (variance) between the two groups; as for the interpretation, the important aspect is the combination rather than individual elements, in contrast to univariate models. The details of the method are described below.

#### 2.5.1. Defining the Regions of Interest and Individual Multivariate Features

In order to derive regional measures based on the Neuromorphometrics atlas released by “MICCAI 2012 Grand Challenge and Workshop on Multi-Atlas Labeling” (www.neuromorphometrics.com (accessed on 1 February 2022)), we applied the following procedure: (1) we obtained the individual atlas for the modality. Each T1w image of each individual was first segmented into three brain tissue classes (cerebral spinal fluid, gray matter, and white matter),and then labeled using SPM12’s diffeomorphic “geodesic” shooting; (2) we computed the mean values of the regions for each of the three modalities using the individualized atlas. We were able to construct an array for each region Y_reg containing a row for every subject and a column for each modality, with the mean value for that subject in that modality.

#### 2.5.2. Statistical Analysis and Model Estimation

The statistical analysis was based on a Multivariate General Linear Model implemented in Matlab as an SPM12 toolbox (code available in Github see below). Using the multivariate method, we estimated the significance of the association between observations (Y_reg) and the diagnostic outcome encoded in a design matrix X. The Ordinary Least Squares (OLS) procedure yielded a matrix representation of the parameter estimates of B in the multivariate general linear formula. Labels for the different diagnoses were implemented in the analysis. In addition to the diagnostic indicators, we added age, gender, and total intracranial volumes as confounding factors.

#### 2.5.3. Statistical Analysis and Model Testing

To test the significance of the difference between the diagnostic categories, an analysis of the eigenvalues of the variance matrices was used to derive the canonical vector that corresponded to the linear combination that best explained the variance of the data, together with an F-test of the Wilks lambda (see [30]). Detailed data on the analysis code and results are available at https://zenodo.org/record/5865628 (accessed on 1 February 2022).

## 3. Results

### 3.1. Demographic and Clinical Characteristics

The two patient groups did not differ significantly in their clinical and demographic features (Table 1). 

### 3.2. Mass Multivariate Analysis Results

**Figure 1:**
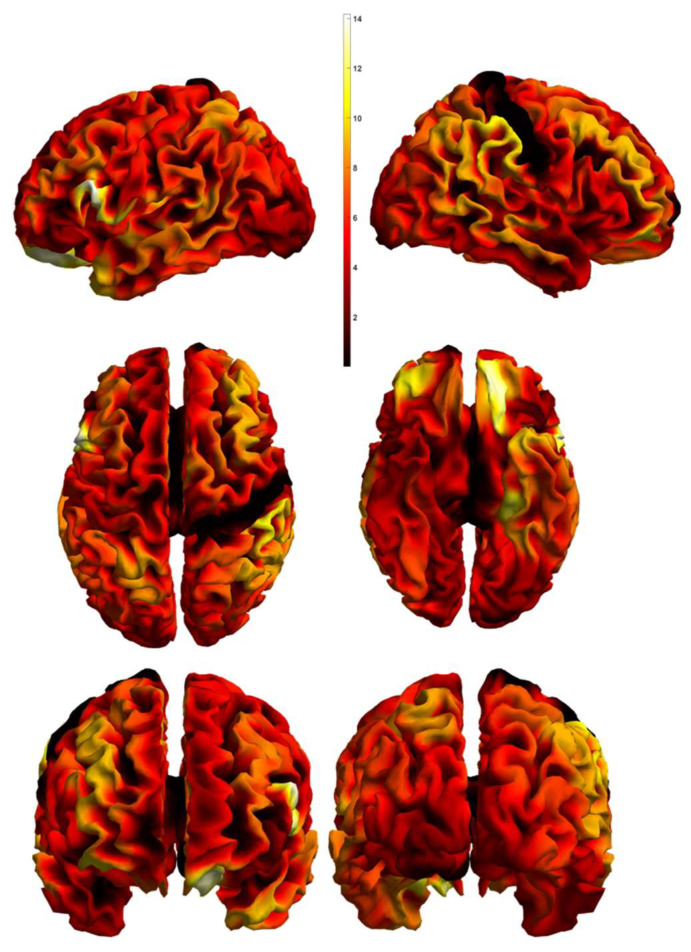
Multivariate Statistical Maps. 3D mesh projections of the Chi statistics testing the significance of the differences between the diagnostic groups. The top row shows the left and right views, the middle row shows the top and bottom view, and the bottom row shows the front and rear views.

We extracted the brain regions from an atlas, presented in the previous section, and implemented them in each modality. Following this, we conducted a Mass Multivariate Analysis. This resulted in the definition of 119 regions with a calculated *p* value, chi-square value, and canonical vectors. Canonical vectors refer to the three modalities—CV 1 to the functional MRI data, CV 2 to the resting-state fMRI data, CV 3 to the structural MRI data. In Table 2, we present 44 regions out of 119 with a range of *p* < 0.01–*p* < 0.05. Moreover, in Figure 1, the Chi-statistics between the tested groups is presented.

According to our methodology, the regions with highest weight appeared to be as follows: left planum polare, left opercular part of the inferior frontal gyrus, medial orbital gyrus, posterior insula, and parahippocampal gyrus (See Table 2). These regions demonstrated a level of significance *p* < 0.01, corrected. However, the *p*-values of the PP demonstrated a level of significance below 0.001.

The differences between the two groups in the planum polare were predominantly driven by the functional imaging modalities (functional and resting state) and a negative weighting for anatomical measures. The canonical vector for the left lateral orbitofrontal cortex (OFC) demonstrated the highest discriminative value between schizophrenia and depression. In addition, the analysis showed resting-state and task-related driven dysfunction of the medial orbital gyrus. The contribution of the parahippocampal region in our analysis had the most significant differentiating capacity in its resting-state activity

**Figure 2:**
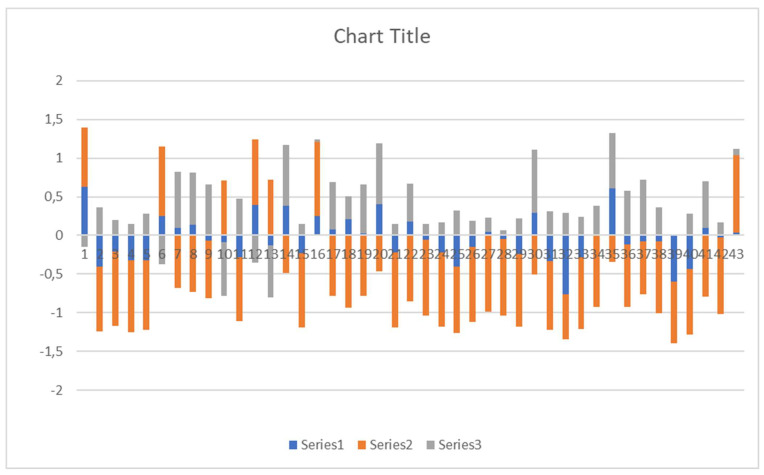
Canonical vectors according to their weights in the three modalities (blue—task fMRI, orange—resting fMRI, grey—structural MRI). The numbers correspond to the different regions in Table 2.

Figure 2 presents all of the above regions according to their canonical vectors and their contribution in each modality. It depicts how the three modalities correspond in the region. This will be discussed in the following section for the first five regions.

**Figure 3:**
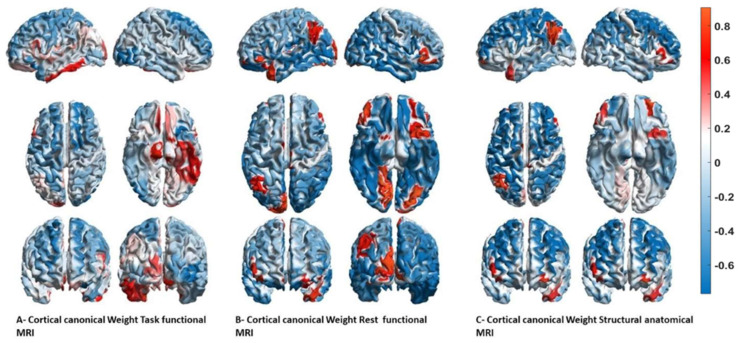
Canonical weighting for the three modalities in explaining different diagnostics. These figures represent the projection of the mass multivariate MLM weights onto a 3D cortical mesh for each modality. (**A**) Task fMRI, (**B**) rest fMRI, and (**C**) anatomical structural MRI. The top row shows the left and right views, the middle row shows the top view, and the bottom row shows the front and rear views. Blue represents a negative contribution, and red represents a positive contribution (please note that the signs are arbitrary, as with any multivariate decomposition).

The results from the MLM are presented in Figure 3 with their canonical weighting for the three modalities (structural MRI, functional MRI, resting-state MRI).

## 4. Discussion

Using the Mass Multivariate Method on structural, resting-state, and task-related fMRI data from two groups of patients with schizophrenia and depression, we were able to define several brain regions with significant relevance to differential diagnosis. Those regions included left planum polare, left OpIFG, MOrG, posterior insula (PIns), and PHG. Our interpretation of these findings is given below. 

The planum polare is a part of the superior temporal gyrus, located just anterior to the Heschl’s gyrus, and is considered to encompass two of the five non-primary auditory areas (the anterior and the medial area). The enhanced connectivity between the thalamus and the right planum polare (among other regions) has been demonstrated to be associated with a higher proprioceptive drift (bottom-up processing) during exteroception in HC [31]. Meanwhile, increased activity in the left planum polare has been related to attentional processes during audiovisual dialogues [32]. One of the major symptoms in depression is the subjective perception of reduced attention and memory as well as a decreased ability to participate in a conversation. Our results showing the significance of the left planum polare for differentiation signatures is in line with the phenomenological presentation of disrupted self-perception in depression.

The involvement of the auditory cortex in the pathophysiology of schizophrenia has been demonstrated in both structural and functional MRI studies [33,34,35,36,37]. A progressive reduction of gray matter volume of the superior temporal gyrus (including planum polare and planum temporale) has been detected in a longitudinal study of patients with ultra-high risk for psychosis and first episode [38]. On the other hand, a smaller GMV of the PP was found in patients with bipolar depression compared to patients with unipolar depression [39].

In healthy individuals, the acute administration of a dopamine precursor (L-DOPA) was followed by a reduction of functional connectivity between the right anterior insula and the left auditory cortex planum polare [40]. In addition, there was a strong positive correlation between the score on a scale assessing psychotic-like experiences and the functional connectivity between right AI and planum polare. The findings suggest that psychotic-like experiences are associated with a dopamine-induced disruption of auditory input to the salience network (SN), which may lead to aberrant attribution of salience. Thus, the existing evidence of the involvement of planum polare in the pathophysiology of psychosis points to both functional and structural abnormalities. Our results highlight a contribution with a slight prevalence of resting-state rather than task-related fMRI function of this area to the differential diagnosis of schizophrenia and affective disorders.

The orbital part of the inferior frontal gyrus is known as Brodmann area 47, the major functions of which are related to language processing and comprehension as well as emotion recognition [41]. Along with Brodmann area 45, it is part of the semantic network. The latter is demonstrated to be dysfunctional in schizophrenic patients, where the disruption is found to correlate with the severity of formal thought disorder [42]. On the other hand, delusion misattribution was associated with cortical thickness in Brodmann’s area (BA) 11/47 in first-episode psychosis [43]. Moreover, the duration of illness was inversely related to regional gray matter volume in the left inferior frontal gyrus, more specifically, BA 11/47 [44].

On the other hand, depression has been characterized by increased functional connectivity of the lateral orbitofrontal cortex (OFC)—BA 47/12 (non-reward/punishment system)—with the precuneus (involved in the sense of self and agency) and the angular gyrus (involved in language) related to the negative sense of the self and of self-esteem [45]. Similar findings of disturbed function of the lateral OFC led to the formulation of the so-called non-reward attractor theory of depression [46]. According to it, the non-reward system is more easily triggered and maintains its attractor-related firing for longer, which leads to negative cognitive states, having positive feedback to the lateral OFC non-reward system once again [45]. In our study, the resting-state component of the canonical vector for the left lateral OFC demonstrated the highest discriminative value between schizophrenia and depression, which is in line with the abundant data on connectivity disruption of this brain area in depression.

The orbitofrontal cortex (OFC) has been associated with complex physiological processes such as reward-based decision making, regulation of the affect, etc. [47,48]. Furthermore, connectivity patterns of the medial and lateral OFC have been related to goal-directed behavior, and existent aberrations in the anatomical or functional connections have been implicated in various mental disorders such as MDD and obsessive-compulsive disorder (OCD) [49]. In relation to MDD, in a previous study utilizing voxel-based morphometry analysis, our research group found a significant decrease of the GMV in the right medial orbital gyrus (MOG) in depressed patients as compared to healthy individuals [9]. In addition, GMV reduction in the right MOG and the right parahippocampal region was found in schizophrenic patients with persistent negative symptoms (PNS) as compared to non-PNS patients and healthy controls [50]. Spalletta et al. also implicated impaired white matter structural integrity of the MOG in the pathophysiology of schizophrenia [51], thus providing further substantiation for the role of this locus in the pathophysiology of these severe conditions.

In terms of functional aberrations, increased functional connectivity between the OFC and the medial prefrontal cortex, as well as hyperconnectivity between the amygdala and the hippocampus, were demonstrated in individuals with a paranoid syndrome compared to HC [52]. Fronto-limbic circuitry impairment has also been a consistent finding in MDD [53]. Therefore, our findings, suggesting a resting-state and task-related driven dysfunction of the MOG, are concordant with the hitherto acquired knowledge on the pathophysiology of MDD and SCH.

Another corresponding result is the contribution of the parahippocampal region, which in our analysis has the most significant differentiating capacity in its resting-state activity. The parahippocampal gyrus (PHG) encompasses a large part of the medial temporal lobe and is a known node of the limbic system. Even though its precise functions are not yet fully understood, evidence suggests that the PHG is an integral factor in cognition, specifically in memory encoding—e.g., object location [54], events, facts [55]. In addition, it is hypothesized that the PHG is a moderating hub of the DMN in the medial temporal lobe [56]. According to Razi et al., in patients with chronic schizophrenia there is a significant GMV reduction in the left parahippocampal gyrus in comparison with healthy controls [57]. Moreover, another study determined that parahippocampal deactivation precedes auditory hallucinations in patients with SCH [58,59,60]. According to our methodology, PHG is more significant in the resting state modality than in the task-based fMRI.

The posterior insula primarily has connections with posterior temporal, parietal, and sensorimotor areas and retains somatosensory representations of touch to one’s own body in contrast with other somatosensory areas, being activated by the sight of touch [61]. PIns is linked to the perception of pain and temperature. In addition, during auditory perception, the responses of PIns are similar to the ones registered in Heschl’s gyrus. The posterior insula has functional connectivity with the somatosensory cortices, posterior and middle cingulate, and temporo-parietal regions, suggesting a more predominant role in somatosensory recognition, homeostatic processing, and monitoring of inner bodily states [62]. A meta-analysis of gray matter changes in schizophrenia revealed a medium-sized reduction in insula volume, of greatest magnitude in the anterior subregion compared to the posterior subregion [63]. In addition, in our analysis, we found that PIns has higher significance during resting-state fMRI.

In first-episode schizophrenia (FES) patients, aberrant differential activation in the posterior insula for first-person tactile experiences and observed affective tactile stimulations was found, suggesting that social perception in FES at a pre-reflective level is characterized by disturbances of self-experience, including impaired multisensory representations and self–other distinction [64]. In addition, the PIns showed reduced functional coupling with the posterior cingulate cortex (PCC) and the postcentral gyrus and increased functional interactions with the anterior insula. These results suggest an imbalance in the processing between internally and externally guided information and its abnormal integration with self-referential processing as mediated by the PCC [65].

## 5. Limitations of the Study

This study has several limitations. The first is related to the small sample size of each of the included groups. This is also characteristic of most pro-innovative research on medical biomarkers related to diagnosis and prognosis of disease, which initially report small groups [66]. Nonetheless, the predictive power of these biomarkers has been later confirmed in larger cohorts [67].

In addition, the current neuro-psychiatric research based on big data collection has not delivered the expected progress in the field. Therefore, we aspire to use as many tools as possible to define small but homogenous cohorts and to achieve incremental validity, which entails additional measures to be intensely applied to small samples in order to achieve increased evidence strength [68]. Another limitation is the arbitrary sign defining the values of the different modalities’ contribution to diagnosis in the relevant figure (Figure 2). Although it is evident that for most of the regions the contribution of resting-state MRI was opposite to the contribution of structural and t-r functional MRI, further investigations are needed to specify the extent to which those canonical vectors have the potential to identify specific diagnostic classes.

## 6. Conclusions

This study delivers evidence in support of multimodal neuroimaging approaches that may add incremental validity to psychiatric diagnosis. Data acquired with structural, resting-state, and task-related functional MRI modalities are limited and sometimes controversial as to their potential integration as biomarkers of diagnosis. In multivariate integration, one can identify robust components based on the evidence accumulated along the different imaging modalities, even if they are individually highly variable.

Further studies need to consider and implement a convergent cross-validation of clinical measures incorporated in multivariate analysis to discriminate depressive disorders from other clinical conditions and healthy states. Biological signatures of disease on the level of neuroimaging in psychiatry may underpin new nosological entities in psychiatry, when considered on a broader basis of nomothetic networks [1,2,69].

## Figures and Tables

**Table 1:** Demographic and clinical characteristics of the participants.

	Schizophrenia Patients (*n* = 19)	Depressed Patients (*n* = 25)	Statistical Significance
Age (mean ± SD)	39.3 ± 14.8	44.2 ± 12.1	0.231 ^a^
Sex (M/F)	9/10	9/16	0.542 ^b^
Education (years)	13.5 ± 2.8	14.1 ± 3.5	0.548 ^a^
Age at onset (years)	27.1 ± 9.1	33.8 ± 12.4	0.139 ^a^
Illness duration (months)	142.8 ± 121.6	121.8 ± 84.5	0.505 ^a^
Episode duration (weeks)	15.4 ± 14.1	11.9 ± 10.4	0.403 ^a^
MADRS score	-	30.5 ± 6.0	-
PANSS score	58.5 ± 13.6	-	-
CGI-S score	4.14 ± 0.66	4.18 ± 0.75	0.891 ^a^

SD—Standard Deviation, MADRS—Montgomery–Åsberg Depression Rating Scale, PANSS—Positive and Negative Syndrome Scale, CGI-S—Clinical Global Impression—Severity, ^a^ Independent samples *t*-test, ^b^ χ^2^-test.

**Table 2:** Significant regions and their canonical vectors.

Regions		*p*-Value	Chi Statistics	Canonical Vector		
				CV1	CV2	CV3
1	Left planum polare	0.0008	16.7299	0.6319	0.7613	−0.1453
2	Left opercular part of the inferior frontal gyrus	0.0022	14.5809	−0.4026	−0.8402	0.3633
3	Left medial orbital gyrus	0.0039	13.3569	−0.2124	−0.9564	0.2005
4	Left posterior insula	0.0077	11.9015	−0.3233	−0.9339	0.1530
5	Left parahippocampal gyrus	0.0092	11.5353	−0.3229	−0.9040	0.2800
6	Right lateral orbital gyrus	0.0121	10.9252	0.2551	0.8937	−0.3690
7	Right supramarginal gyrus	0.0134	10.7141	0.0964	−0.6804	0.7265
8	Right anterior orbital gyrus	0.0169	10.2093	0.1428	−0.7306	0.6677
9	Right supplementary motor cortex	0.0197	9.8746	−0.0688	−0.7482	0.6599
10	Left supplementary motor cortex	0.0203	9.8031	−0.0859	0.7082	−0.7007
11	Left superior temporal gyrus	0.0208	9.7535	−0.2775	−0.8334	0.4779
12	Left temporal pole	0.0211	9.7250	0.3911	0.8486	−0.3563
13	Left anterior orbital gyrus	0.0238	9.4530	−0.1262	0.7232	−0.6790
14	Right middle frontal gyrus	0.0243	9.4139	0.3836	−0.4898	0.7829
15	Left Amygdala	0.0263	9.2404	−0.2334	−0.9612	0.1474
16	Left frontal operculum	0.0267	9.2025	0.2479	0.9685	0.0226
17	Right angular gyrus	0.0274	9.1444	0.0716	−0.7811	0.6202
18	Right middle temporal gyrus	0.0277	9.1216	0.2095	−0.9312	0.2982
19	Left superior frontal gyrus medial segment	0.0285	9.0578	0.0298	−0.7775	0.6282
20	Left superior parietal lobule	0.0305	8.9095	0.4082	−0.4644	0.7859
21	Left Hippocampus	0.0307	8.9001	−0.2239	−0.9630	0.1502
22	Right superior temporal gyrus	0.0332	8.7205	0.1746	−0.8503	0.4964
23	Right posterior insula	0.0341	8.6677	−0.0524	−0.9878	0.1469
24	Left central operculum	0.0348	8.6178	−0.2178	−0.9620	0.1649
25	Left fusiform gyrus	0.0350	8.6045	−0.4066	−0.8550	0.3219
26	Left middle cingulate gyrus	0.0351	8.5999	−0.1485	−0.9698	0.1935
27	Left medial frontal cortex	0.0366	8.5070	0.0482	−0.9819	0.1833
28	Right parietal operculum	0.0378	8.4347	−0.0461	−0.9966	0.0686
29	Right middle cingulate gyrus	0.0388	8.3761	−0.2403	−0.9456	0.2196
30	Left middle frontal gyrus	0.0395	8.3418	0.2894	−0.5013	0.8154
31	Left gyrus rectus	0.0403	8.2928	−0.3306	−0.8902	0.3133
32	Left entorhinal area	0.0406	8.2803	−0.7626	−0.5786	0.2894
33	Left posterior cingulate gyrus	0.0441	8.0939	−0.2762	−0.9312	0.2378
34	Left middle temporal gyrus	0.0451	8.0429	−0.0043	−0.9256	0.3785
35	Right superior frontal gyrus	0.0453	8.0345	0.6047	−0.3385	0.7209
36	Left anterior cingulate gyrus	0.0471	7.9465	−0.1187	−0.8082	0.5768
37	Right anterior cingulate gyrus	0.0493	7.8452	−0.0797	−0.6843	0.7249
38	Right medial orbital gyrus	0.0501	7.8105	−0.0785	−0.9286	0.3626
39	Left Basal Forebrain	0.0508	7.7787	−0.6022	−0.7983	0.0046
40	Right gyrus rectus	0.0513	7.7589	−0.4299	−0.8586	0.2792
41	Right superior frontal gyrus medial segment	0.0521	7.7212	0.1002	−0.7965	0.5963
42	Right CO central operculum	0.0526	7.7016	−0.0305	−0.9853	0.1684
43	Right medial frontal cortex	0.0544	7.6275	0.0393	0.9953	0.0889

## Data Availability

Data is available upon request.
